# A Rare Case of Disseminated Pyogenic Gonococcal Infection in an Immunocompetent Woman

**DOI:** 10.1155/2016/9629761

**Published:** 2016-12-27

**Authors:** Iordanis Romiopoulos, Athina Pyrpasopoulou, Anna Varouktsi, Elisavet Simoulidou, Konstantina Kontopoulou, Ekaterini Karantani, Vivian Georgopoulou, Konstantinos Kitsios, Apostolos Mamopoulos, Charalampos Antachopoulos, Asterios Karagiannis, Emmanuel Roilides

**Affiliations:** ^1^Infectious Diseases Unit, 3rd Department of Pediatrics, Faculty of Medicine, Aristotle University School of Health Sciences, Hippokration General Hospital, Thessaloniki, Greece; ^2^2nd Propaedeutic Department of Internal Medicine, Faculty of Medicine, Aristotle University School of Health Sciences, Hippokration General Hospital, Thessaloniki, Greece; ^3^Laboratory of Microbiology, Gennimatas General Hospital, Thessaloniki, Greece; ^4^Laboratory of Microbiology, Hippokration General Hospital, Thessaloniki, Greece; ^5^Laboratory of Radiology, Hippokration General Hospital, Thessaloniki, Greece; ^6^Department of Medicine, Gennimatas Hospital, Thessaloniki, Greece; ^7^3rd Department of Obstetrics and Gynecology, Faculty of Medicine, Aristotle University School of Health Sciences, Hippokration General Hospital, Thessaloniki, Greece

## Abstract

We present a case of previously healthy, immunocompetent, 41-year-old woman who developed systemic inflammatory response syndrome secondary to* Neisseria gonorrhoeae* bacteremia. Clinical course was complicated by the simultaneous formation of multiple muscular abscesses, epidural abscess, and septic spondylodiscitis. The patient responded well to prolonged ceftriaxone treatment and was released 10 weeks after initial admission. Spinal lesions and/or pyomyositis individually constitute rare complications of disseminated gonococcal infection. This case, combining both manifestations, is to our knowledge unique. Apropos, diversity of the clinical presentation, and therapeutic challenges for this historical disease are discussed for the practicing physician.

## 1. Introduction

Gonococcal disease is generally asymptomatic or mildly symptomatic [1]. Disseminated disease and distal septic complications such as spinal abscesses and pyomyositis have been very rarely reported [2]. We describe the case of a female, who developed disseminated disease accompanied by severe systemic inflammatory response syndrome (SIRS), pyomyositis, and spinal lesions including septic spondylodiscitis and epidural abscess with neurological symptoms. To our knowledge, this is the first case reported of such extensive complications including spinal lesions and pyomyositis.

## 2. Case Presentation

A 41-year-old woman was referred to the 3rd Department of Obstetrics and Gynecology of Hippokration General Hospital for further investigation and treatment of pyogenic pelvic inflammatory disease. The patient's past history included bowel obstruction at the age of 8 months with no more data available and 3 caesarian sections; she was taking no chronic medication.

Symptoms started 9 days before referral with intense low back pain of abrupt onset for which she had consulted an orthopedic surgeon and had been prescribed nonsteroidal anti-inflammatory drugs (NSAIDS) without response. Two days later (day 1) the patient was admitted to the Department of Medicine, Gennimatas Hospital, with fever (38.2°C) and abdominal pain, localized in the right upper quadrant and the epigastrium. Laboratory tests revealed leukocytosis (WBC 14,900/*μ*L, 96% neutrophils), thrombocytopenia (60,000/*μ*L), markedly elevated C-reactive protein (330 mg/L), and mildly affected liver chemistry (SGOT 60 U/L, SGPT 84 U/L, *γ*GT 104 U/L, ALP 160 U/L, total bilirubin 2.79 mg/dL, and direct bilirubin 2.62 mg/dL). Initial blood and urine cultures did not grow any pathogen. Despite the normal abdominal ultrasound findings, the patient was treated empirically for potential cholecystitis with intravenous cefoxitin, 1 g tid.

The following day (day 2) the clinical course was further complicated by arthritis of the right shoulder, the right elbow, and the right ankle, which improved with administration of NSAIDS. Abdominal pain, however, worsened involving the lower abdomen, and tenderness of the right thigh developed, which was aggravated by movement. Computed tomography scan of the abdomen revealed no liver and biliary lesions but increased size and calcifications of the uterus, thickening of the perirectal fasciae, formation of perirectal abscesses, collection of fluid in the epidural and presacral space with presence of air, and presence of air within the subcutaneous tissue of the anterior abdominal wall. Given the formation of perirectal abscesses in the setting of a fever with abdominal pain and arthritis, the patient underwent colonoscopy on day 5 and a potential diagnosis of inflammatory bowel disease was excluded. On day 6 the patient had an episode of self-limited vaginal bleeding (her last menstrual cycle being 2 weeks previously). The cervical swab was positive for Gram-negative, coffee bean-shaped diplococci and culture grew* Neisseria gonorrhoeae*. No other pathogens were isolated. Serological tests for HIV and syphilis were negative. The patient denied previously extramarital sexual activity or prior sexually transmitted diseases. She was living with her husband who refused examination for gonorrhoea. Her last sexual intercourse was reported to be 1 month before admission.

On day 7, new blood cultures incubated into the BacT/ALERT automated system (Biomérieux, Marcy-l'Étoile, France) revealed high bacterial load, evidenced by the fact that positive signal was elicited as early as two hours after incubation. A Gram-negative diplococcus was seen on microscopy. The isolate was identified as* N. gonorrhoeae* on the automated system Vitek II (Biomérieux). Due to absence of* N. gonorrhoeae* susceptibility card the antimicrobial susceptibility testing was performed by Kirby Bauer disc diffusion method and revealed susceptibility to ceftriaxone, clindamycin, and ciprofloxacin but resistance to azithromycin.

**Figure 1:**
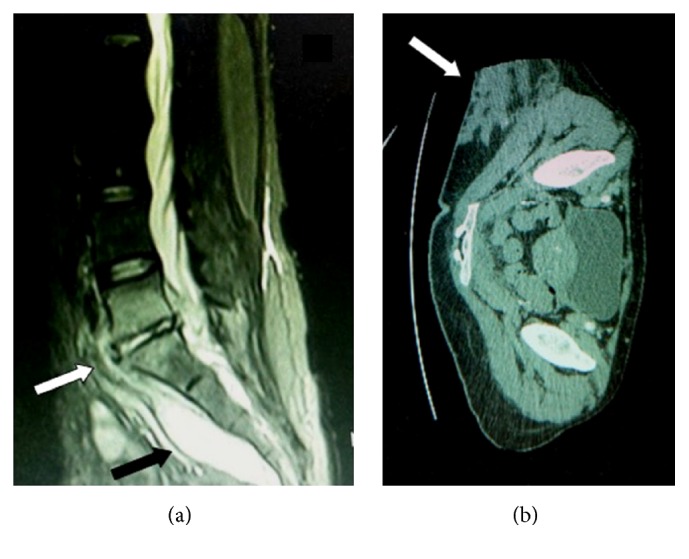
(a) Presacral epidural inflammatory fluid collection (abscess) (black arrow) and adjacent spondylodiscitis that developed at the level of L5-S1 vertebrae (white arrow). Due to its small size the epidural abscess was successfully managed with antibiotics alone. (b) Large abscess of the right buttock (white arrow) that was drained under CT scan guidance.

On the same day the patient was referred to Hippokration Hospital for further evaluation and management of pyogenic pelvic inflammatory disease. At referral she was septic with a temperature of 39.5°C, hypoalbuminemic (2.0 g/dL), and markedly oedematous. A quadruple intravenous antibiotic regimen was initiated (ceftriaxone 2 g q12 h, clindamycin 600 mg q6 h, gentamicin 6 mg/kg q24 h, and azithromycin 600 mg q24 h); the patient gradually responded and became afebrile within a week. On day 8, she developed a hemorrhagic maculopapular rash in her lower extremities, painful palpable lumps in the right thigh, and shin and splinter hemorrhages. Endocarditis was excluded by transesophageal echocardiography. Magnetic resonance imaging confirmed the presence of a presacral epidural inflammatory collection with associated septic spondylodiscitis of the 5th lumbar and 1st sacral vertebrae and abscesses of multiple muscles (pyomyositis) including psoas, gluteus maximus, and quadratus femoris, the largest among them measuring 12.1 cm × 6.7 cm (Figures 1(a) and 1(b)).

On day 20, this large abscess was drained and 160 mL of purulent fluid was removed under CT scan guidance. Gram stain and cultures of the fluid were negative. In the setting of a disseminated gonococcal disease with multiple complications, the possibility of an underlying immunodeficiency was investigated by quantitative analysis of immunoglobulins and components of complement, with normal findings. The antibiotic regimen was gradually deescalated to only intravenous ceftriaxone, which was continued for 6 weeks. CRP levels normalized very slowly and finally the patient was discharged with oral ciprofloxacin 500 mg bid for a month.

## 3. Discussion

The case described in this report has several unusual and interesting features. The clinical course of the infection in an otherwise immunocompetent woman escalated from asymptomatic gonorrhoea to disseminated gonococcal disease. At this point, Fitz-Hugh-Curtis syndrome, also known as acute perihepatitis, characterized by inflammation of the peritoneum and the perihepatic tissues [3] was considered in relation to gonococcal disease. The syndrome can be underdiagnosed because of subtle CT liver and peritoneal findings. Of note, cefoxitin used to treat probable cholecystitis did not prove efficient against gonococcal syndrome although it is considered in vitro active. This underlies the fact that the efficacy of an antibiotic is not determined only by antibiotic pharmacodynamics but is based on pharmacokinetics/pharmacodynamics index, which was possibly not fulfilled in this case [4]. On the other hand, although azithromycin was resistant in vitro, it was included in the therapeutic regimen at least for a potential broader and enhanced activity given the rapid evolution of the clinical condition. Septic arthritis is a well-characterized late complication of the bacteremic stage of the disease; the spinal vertebrae however become affected very rarely [5]. Our patient may have developed this complication due to preexisting degenerative lesions of the spine and the adjacent inflammatory fluid collection that developed in the course of the disease.

Disseminated gonococcal disease is generally rare (1–3%) and has been reported mostly in immunocompromised patients with complement or other immunological deficiencies [6–9]. There are only few published cases of disseminated pyogenic gonococcal infection either as spondylitis [5] or as pyomyositis [10].

The prevalence of gonococcal pyomyositis is extremely low. In 1992, a review included 100 cases of pyomyositis in North America over a period of 20 years, of which no case was gonococcal [11]. Gonococcal pyomyositis has been reported in 6 patients [10, 12–16]. The involved muscular sites were thigh and calf [10], biceps brachii [12, 14], and obturator internus [13, 15]. On the other hand, an axial skeleton involvement in the setting of a disseminated gonococcal infection is maybe an even more rare complication. In 1976, Seruzier et al. published probably the first case of gonococcal spinal infection in a 47-year-old male with spondylodiscitis at the level of 9th and 10th thoracic vertebra [17]. In 2004, Van Hal and Post reported a thoracic epidural mass at 6th to 7th vertebra [18] and more recently Low et al. reported a case of gonococcal spinal epidural abscess extended from the 6th cervical to the 2nd thoracic vertebra without cord compression [5].

Our case is unique because, to our knowledge, disseminated gonococcal disease of an excessively suppurative form affecting both spine and multiple muscles has not been previously reported. The intensity of the symptomatology in this otherwise healthy individual, the complications that she developed, and the delayed response to appropriate treatment necessitated increased medical care and prolonged hospitalization.

*N. gonorrhoeae* has always been and probably will remain a major health problem, which rarely may involve difficult-to-manage spine and muscular septic complications. Awareness of the diversity of the clinical presentation and therapeutic challenges for this historical disease remains therefore important for the practicing physician.

## References

[1] Cook P. A., Evans-Jones J., Mallinson H. (2014). Comparison of patients diagnosed with gonorrhoea through community screening with those self-presenting to the genitourinary medicine clinic. *BMJ Open*.

[2] Belkacem A., Caumes E., Ouanich J. (2013). Changing patterns of disseminated gonococcal infection in france: cross-sectional data 2009–2011. *Sexually Transmitted Infections*.

[3] Muschart X. (2015). A case report with Fitz-Hugh-Curtis syndrome, what does it mean?. *Acta Clinica Belgica*.

[4] Belda W., Velho P. E. N. F., Fagundes L. J., Arnone M. (2007). Evaluation of the in vitro activity of six antimicrobial agents against Neisseria gonorrhoeae. *Revista do Instituto de Medicina Tropical de Sao Paulo*.

[5] Low S. Y. Y., Ong C. W. M., Hsueh P.-R., Tambyah P. A., Yeo T. T. (2012). Neisseria gonorrhoeae paravertebral abscess: case report. *Journal of Neurosurgery: Spine*.

[6] Miller K. E. (2006). Diagnosis and treatment of Neisseria gonorrhoeae infections. *American Family Physician*.

[7] Amir O., Nguyen V. D., Barnett B. J. (2003). Acute human immunodeficiency virus infection presenting as disseminated gonococcal infection. *Southern Medical Journal*.

[8] Keiser H. D. (1997). Recurrent disseminated gonococcal infection in a patient with hypocomplementemia and membranoproliferative glomerulonephritis. *Journal of Clinical Rheumatology*.

[9] Ellison R. T., Curd J. G., Kohler P. F., Reller L. B., Judson F. N. (1987). Underlying complement deficiency in patients with disseminated gonococcal infection. *Sexually Transmitted Diseases*.

[10] Jitmuang A., Boonyasiri A., Keurueangkul N., Leelaporn A., Leelarasamee A. (2012). Gonococcal subcutaneous abscess and pyomyositis: a case report. *Case Reports in Infectious Diseases*.

[11] Christin L., Sarosi G. A. (1992). Pyomyositis in North America: case reports and review. *Clinical Infectious Diseases*.

[12] Swarts R. L., Martinez L. A., Robson H. G. (1981). Gonococcal pyomyositis. *Journal of the American Medical Association*.

[13] Gurbani S. G., Cho C. T., Lee K. R., Powell L. (1995). Gonococcal abscess of the obturator internal muscle: use of new diagnostic tools may eliminate the need for surgical intervention. *Clinical Infectious Diseases*.

[14] Haugh P. J., Levy C. S., Hoff-Sullivan E., Malawer M., Kollender Y., Hoff V. (1996). Pyomyositis as the sole manifestation of disseminated gonococcal infection: case report and review. *Clinical Infectious Diseases*.

[15] Birkbeck D., Watson J. T. (1995). Obturator internus pyomyositis. A case report. *Clinical Orthopaedics and Related Research*.

[16] Owino N. O., Goldmeier D., Wall R. A. (1981). Gonococcal septicaemia presenting as a subcutaneous abscess. *British Journal of Venereal Diseases*.

[17] Seruzier E., Blanquart F., Lemeulant J. F., Deshayes P. (1976). Gonococcal spondylodiscitis. *La Nouvelle Presse Medicale*.

[18] Van Hal S. J., Post J. J. (2004). An unusual cause of an epidural abscess. *Medical Journal of Australia*.

